# Physical activity and cognitive performance: cross-sectional analysis of the Brazilian longitudinal study of adult health (ELSA-Brazil)

**DOI:** 10.1016/j.clinsp.2026.101076

**Published:** 2026-09-01

**Authors:** Amanda M. Pádua, Claudia K. Suemoto, Patrícia Fonseca Estrada, Marcos R.N. Cavalcante, Alessandra C. Goulart, Paulo Andrade Lotufo, Itamar de Souza Santos, Isabela M. Bensenor

**Affiliations:** a Faculdade de Saúde Pública, Universidade de São Paulo, São Paulo, SP, Brazil; b Division of Geriatrics, Faculdade de Medicina da Universidade de São Paulo, São Paulo, SP, Brazil; c Faculdade de Medicina da Universidade de São Paulo, São Paulo, SP, Brazil; d Clinical and Epidemiological Research Center, University Hospital, Universidade de São Paulo, São Paulo, SP, Brazil; e Department of Epidemiology, Faculdade de Saúde Pública, Universidade de São Paulo, São Paulo, SP, Brazil; f Department of Internal Medicine, Faculdade de Medicina da Universidade de São Paulo, São Paulo, SP, Brazil

**Keywords:** Leisure-time physical activity, Commuting physical activity, Cognitive performance, Cohort studies

## Abstract

**Introduction:**

Evidence on the relationship between Leisure-Time Physical Activity (LTPA), Commuting Physical Activity (CPA), and cognitive performance is inconsistent.

**Objective:**

To examine the associations between LTPA, CPA, and cognitive performance in adults from the Brazilian Longitudinal Study of Adult Health (ELSA‒Brazil).

**Methods:**

This cross-sectional analysis included 13,340 participants (median age 51 years; 54.5% women). Physical Activity (PA) was assessed using the long-form International Physical Activity Questionnaire, including LTPA and CPA. Cognitive performance was measured by word memory, verbal fluency, and the Trail Making Test Part B. Z-scores were computed for each test, and a global cognitive score was derived as their mean. Linear regression models estimated associations between PA levels and cognitive performance after multivariable adjustment, including occupational status. Analyses were stratified by sex and conducted in the overall sample.

**Results:**

LTPA inactivity was associated with lower scores for verbal fluency (β = -0.07; 95% CI: -0.10 to -0.04), executive function (β = -0.10; 95% CI: -0.13 to -0.06), and global cognition (β = -0.06; 95% CI: -0.08 to -0.03). CPA inactivity showed associations in the opposite direction, with higher executive function (β = 0.08; 95% CI: 0.05 to 0.12) and memory in men (β=0.05; 95% CI: 0.001 to 0.10). After adjustment for occupational status, CPA associations attenuated and lost significance.

**Conclusion:**

In this cross-sectional study, the relationship between PA and cognition differed by domain. LTPA was associated with better cognitive scores, while CPA showed non-favorable or null associations after adjustment.

## Highlights

• Leisure-time inactivity associated with lower overall cognitive performance.
• Inactive commuting linked to better executive function in both sexes.
• Cognitive effects of physical activity vary by activity domain.
• Consistent LTPA–cognition associations observed in men and women.

## Introduction

Physical Activity (PA) is widely recognized as a fundamental pillar of public health, with the World Health Organization (WHO) recommending at least 150 minutes of moderate-intensity activity or 75 minutes of vigorous-intensity activity per week, or an equivalent combination of both.^1^ While these guidelines have remained broadly consistent over time, recent updates emphasize the critical importance of total PA, regardless of the specific type, frequency, or intensity.^2^ This broader perspective highlights the adaptability of PA recommendations to diverse lifestyles and individual preferences in achieving health benefits.

Despite these disseminated guidelines, further investigation is needed into how Leisure-Time Physical Activity (LTPA) and Commuting Physical Activity (CPA) uniquely impact health outcomes, particularly given their different demographic and socioeconomic associations. More recently, a body of evidence highlights the benefits of PA on cardiovascular risk factors, cardiovascular disease, and mortality.^3^^,^^4^ However, conflicting results have been identified when comparing the effects of PA during leisure or commute on specific outcomes, such as hypertension^5^, ^6^, ^7^, ^8^ or diabetes.^9^^,^^10^

Leisure-Time Physical Activity (LTPA) is often associated with a lower prevalence of outcomes such as hypertension, as observed in cross-sectional^11^ and prospective studies.^12^^,^^13^ In contrast, CPA has shown mixed associations with hypertension in several global studies, with some indicating a positive association,^5^^,^^14^^,^^7^ while recent prospective findings have suggested a protective effect.^8^ Furthermore, LTPA and CPA patterns differ significantly by sex,^15^, ^16^, ^17^ highlighting the importance of considering this factor to understand their distinct impacts on health outcomes. The increasing global prevalence of cognitive impairment and dementia directly correlates with population aging.^14^^,^^18^ Although studies have established the benefits of PA on cognitive performance,^19^ dementia,^20^^,^^21^ or both,^22^ research specifically investigating the relationship between the domain of PA practice (e.g., leisure time vs. commuting) and cognitive performance remains limited.^23^, ^24^, ^25^ Furthermore, few of these investigations have simultaneously analyzed both domains in a single cohort of middle-aged and older men and women.^24^^,^^25^

Therefore, this study aims to investigate the cross-sectional association between PA performed during leisure time and commuting and cognitive test performance, using baseline data from participants in the Brazilian Longitudinal Study of Adult Health (ELSA ‒ Brazil).

## Materials and methods

### Design and population study

The Brazilian Longitudinal Study of Adult Health (ELSA‒Brazil) is a prospective cohort study of 15,105 civil servants aged 35 to 74 years from six Brazilian cities. The primary objective is to identify determinants of Cardiovascular Disease (CVD) and diabetes. Detailed descriptions of the study design and methodology have been previously published.^26^, ^27^, ^28^, ^29^ The study protocol was approved by the institutional review boards of all participating centers and conducted in accordance with the Declaration of Helsinki. All participants provided written informed consent.^26^

Baseline data were collected between 2008 and 2010. Participants underwent structured interviews and clinical evaluations. Participants underwent structured interviews and clinical evaluations conducted by trained personnel using standardized protocols under strict quality control.^26^

The protocol of the study was approved by each institutional ethics committee (approval numbers 0017.1.069.000-06 BA, 08109612.7.20035060 ES, 0186.1.203.000-06 MG, 0017.1.069.000-06 RS, 0058.0.011.000-07 RJ, and 0016.1.198.000-06 SP for the baseline assessments and 30614714.8.1001.5030 BA, 08109612.7.20035060 ES, 0186.1.203.000-06 MG, 48608515.5.1001.5327 RS, 56021516.0.1001.5240 RJ, and 08109612.7.1001.0076 SP for the follow-up assessments). The study was conducted in accordance with the Declaration of Helsinki, and all participants signed an informed consent.

This study was reported in accordance with the Strengthening the Reporting of Observational Studies in Epidemiology (STROBE) guidelines.

### Study participants

**Fig. 1:**
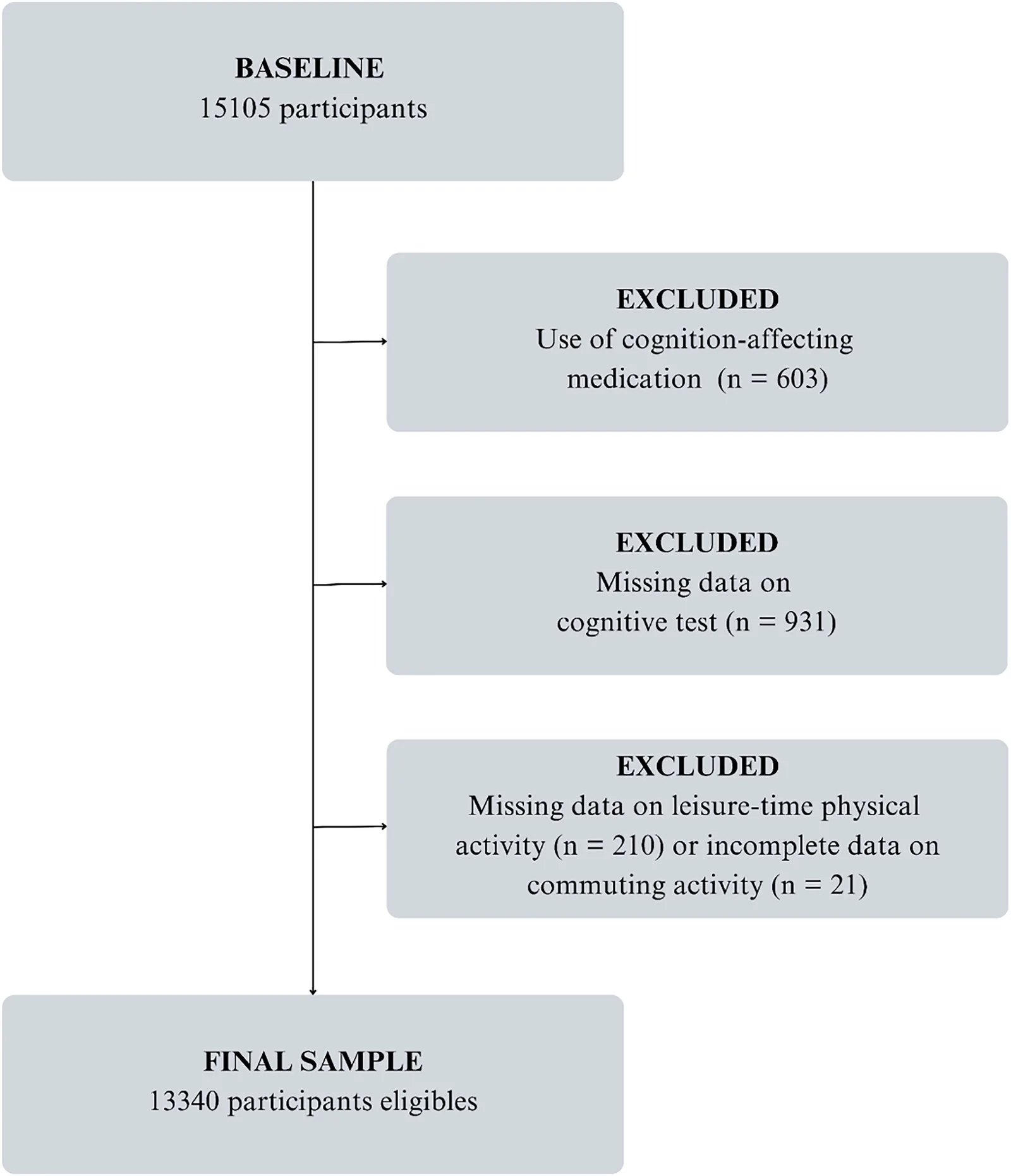
Flowchart of participant selection for the present analysis.

From the original 15,105 participants, the authors excluded individuals using cognition-affecting medications (n = 603), those with missing data on at least one cognitive test (n = 931), those lacking LTPA information (n = 210), and those with incomplete CPA data (n = 21). The final analytic sample included 13,340 individuals (Fig. 1).

### Definition of physical activity

Physical activity was measured using the long-form International Physical Activity Questionnaire (IPAQ), focusing on the LTPA and CPA domains.^30^ This instrument was previously validated in Brazil.^30^ The pattern of PA in its different domains was reported in min per week and calculated by multiplying the weekly rate for the duration of each activity. Issues related to the level of LTPA and CPA were classified according to the recommendations of the WHO.^1^ The categories created were: 1) Physically active (≥150 min per week of moderate activity or ≥ 75 min per week of vigorous activity); 2) Insufficiently active^1^ Any weekly activity below the previous threshold was classified as partly active, and remaining participants were classified as inactive.^1^ Only PA undertaken for at least 10-minutes per week was considered valid.^30^, ^31^, ^32^

### Assessment of cognitive function

The authors assessed cognitive function at baseline with a validated version of the list of words of the Consortium to Establish a Registry for Alzheimer’s Disease (CERAD) with three steps: 1) Immediate recall: participants had to read, and memorize a list of ten words presented three times. The final score was the sum of the words recalled in the three trials 2) Delayed recall: participants were invited to repeat the ten words after a 5-minute time interval when they were occupied in different activities; 3) Word recognition: participants received a card with the ten original words and more ten new words. Participants had to remember the ten original words, ignoring the new words.^33^^,^^34^ The second test is the verbal fluency: for semantic verbal fluency, participants had to name as many animals as they could in 60 seconds, while in phonemic verbal fluency they had to state as many words as they could beginning with the letter “F“ in 60 seconds.^35^^,^^36^ The last test was the Trail Making Test version B (TMT-B), in which trained examiners asked the participants to draw a line alternating between numbers from 1 to 13 in ascending order and letters A to L in alphabetical order as quickly as possible, while the time to perform the test is recorded in seconds.

The authors multiplied the *z*-score of the TMT-B by -1 to ensure that higher scores indicated better cognitive performance, consistent with the interpretation of the other cognitive tests. A global cognitive score was created by summing the standardized *z*-scores of memories, verbal fluency, and the reversed TMT-B. All cognitive tests were administered in a fixed order during a single session, at the beginning of the interview, in a quiet and well-lit room with minimal noise and distractions, when participants were more rested.^37^^,^^38^

### Clinical variables

Diabetes was defined as a prior medical history of diabetes, use of medication to treat diabetes, fasting plasma glucose ≥ 126 mg/dL, 2-hour plasma glucose ≥200 mg/dL, or HbA1C ≥ 6.5% at baseline tests.^39^ The definition of hypertension was based on current use of medications to treat hypertension or on systolic blood pressure ≥140 mmHg and/or diastolic blood pressure ≥ 90 mmHg, validated Omron HEM 705CPINT oscillometric device. Standard techniques were applied for the calculation of Body Mass Index (BMI) and measurement of waist circumference.^40^ Dyslipidemia was defined as a Low-Density Lipoprotein (LDL) cholesterol level ≥ 130 mg/dL or the use of lipid-lowering medication. Levels of LDL-C were calculated using the Friedewald equation, except for cases in which triglyceride levels were > 400 mg/dL, when an enzymatic colorimetric assay (ADVIA 1200) was used.^41^

### Sociodemographic variables

Sociodemographic and health-related questionnaires included information about sex (male, female) and age (continuous and stratified: 35‒44; 45‒54; 55‒64; 65‒74). Race was self-identified according to the categories used in the Brazilian Census: White, Black, Mixed, Indigenous, and Asian descent. These categories reflect the Brazilian Census classification system, which uses self-declared identification based on color or race. The term “Mixed” corresponds to “Pardo” in Portuguese, encompassing individuals of combined African, European, and/or Indigenous ancestry. Education (less than high school, high school and some college, complete college or more), smoking and alcohol status (never, former, current), and average monthly net family income, categorized into three groups: less than US$ 1,245; US$ 1,245 to US$ 3,319; and US$ 3,320 or more. All values are expressed in US dollars, using a conversion rate of 2 Brazilian reais to 1 US dollar, as defined at the study baseline.^28^

### Statistical analysis

Categorical variables were described using frequencies and percentages and compared using Chi-Square tests. Continuous variables were reported as medians with interquartile ranges and compared using the Kruskal-Wallis test.

Cognitive test scores were standardized into *z*-scores by subtracting the sample mean from each participant’s raw score and dividing by the sample standard deviation, so that higher scores indicate better performance across all tests. Associations between LTPA, CPA, and cognitive performance (memory, verbal fluency, executive function, and global cognitive score) were analysed using linear regression models, with the physically active group as the reference category in all models. Analyses were stratified by sex and also conducted in the total sample.

Regression results are presented as β-coefficients with 95% confidence intervals for four models: unadjusted; adjusted for age, race, and education; further adjusted for hypertension, diabetes, smoking, body mass index, and depression; and additionally adjusted for occupational status. Total PA was also evaluated as a combined exposure variable.

Statistical significance was defined as p<0.05. All analyses were conducted using R version 4.5.1 (R Core Team, 2025).

## Results

The study included 13,340 participants, with a median age of 51.0 years (IQR 45.0–58.0); 54.5% were women (n = 7,266).

Baseline characteristics by LTPA (Table 1). Descriptive characteristics differed significantly across LTPA categories (p < 0.05). Physically active participants were younger, more frequently White, and had higher levels of education and income. Among men, 65.0% of those in the active group had completed college or more compared with 47.7% of inactive men (p < 0.001). Similar patterns were observed among women. Active individuals presented lower median BMI and waist circumference and lower prevalence of hypertension, diabetes, and depression (all p < 0.001). No significant differences were observed for dyslipidemia.

**Table 1:** Sociodemographic and clinical characteristics by Leisure-Time Physical Activity (LTPA) levels in men, women, and overall sample.

	Men (n = 6074)				Women (n = 7266)				Overall (n = 13340)			
	Inactive (n=3467)	Insufficiently active (n=837)	Active (n=1770)	p	Inactive (n=4850)	Insufficiently active (n=826)	Active (n=1590)	p	Inactive (n=8317)	Insufficiently active (n=1663)	Active (n=3360)	p
Age (years)	52	52	49	**<0.0001**	51	52	51	**<0.0001**	51	52	50	**<0.0001**
Median (1Q – 3Q)	(45.0 –5 8.0)	(45.0 – 59.0)	(44.0 – 57.0)		(45.0 –57.0)	(46.0 – 59.0)	(45.0 –58.8)		(45.0 – 58.0)	(46.0 – 59.0)	(44.0 –58.0)	
Race (%)				**0.0005**				**<0.0001**				**<0.0001**
White	1774 (51.2)	481 (57.5)	1004 (56.7)		2333 (48.1)	496 (60.1)	957 (60.2)		4107 (49.4)	977 (58.7)	1961 (58.4)	
Mixed	1067 (30.8)	211 (25.2)	500 (28.2)		1373 (28.3)	184 (22.3)	353 (22.2)		2440 (29.3)	395 (23.8)	853 (25.4)	
Black	472 (13.6)	111 (13.2)	196 (11.1)		932 (19.2)	106 (12.8)	188 (11.8)		1404 (16.9)	217 (13.0)	384 (11.4)	
Others	154 (4.4)	34 (4.1)	70 (4.0)		212 (4.4)	40 (4.8)	92 (5.8)		366 (4.4)	74 (4.5)	162 (4.8)	
Education (%)				**<0.0001**				**<0.0001**				**<0.0001**
Less than high school	544 (15.7)	99 (11.8)	122 (6.9)		448 (9.2)	26 (3.1)	55 (3.5)		992 (11.9)	125 (7.5)	177 (5.3)	
High school and some College	1268 (36.6)	252 (30.1)	498 (28.1)		2056 (42.4)	180 (21.8)	356 (22.4)		3324 (40.0)	432 (26.0)	854 (25.4)	
Complete College or more	1655 (47.7)	486 (58.1)	1150 (65.0)		2346 (48.4)	620 (75.1)	1179 (74.1)		4001 (48.1)	1106 (66.5)	2329 (69.3)	
Average family monthly net income (%)				**<0.0001**				**<0.0001**				**<0.0001**
<US$ 1245	948 (27.4)	172 (20.7)	333 (18.9)		1426 (29.5)	123 (14.9)	222 (14.0)		2374 (28.6)	295 (17.8)	555 (16.5)	
US$ 1245 – 3320	1527 (44.1)	325 (39.0)	676 (38.2)		2373 (49.1)	347 (42.1)	695 (43.8)		3900 (47.1)	672 (40.5)	1371 (40,9)	
>US$ 3320	984 (28.5)	336 (40.3)	759 (42.9)		1031 (21.4)	355 (43.0)	669 (42.2)		2015 (24.3)	691 (41.7)	1428 (42.6)	
Smoking (%)				**<0.0001**				**<0.0001**				**<0.0001**
Never	1620 (46.7)	425 (50.8)	1054 (59.6)		2994 (61.7)	524 (63.5)	1047 (65.9)		4614 (55.5)	949 (57.1)	2101 (62.5)	
Past	1258 (36.3)	313 (37.4)	553 (31.2)		1209 (24.9)	215 (26.0)	420 (26.4)		2467 (29.7)	528 (31.7)	973 (29.0)	
Current	589 (17.0)	99 (11.8)	163 (9.2)		647 (13.4)	87 (10.5)	123 (7.7)		1236 (14.8)	186 (11.2)	286 (8.5)	
Alcohol intake				**<0.0001**				**<0.0001**				**<0.0001**
Never	158 (4.6)	27 (3.2)	72 (4.1)		802 (16.6)	94 (11.4)	177 (11.1)		960 (11.6)	121 (7.3)	249 (7.4)	
Past	725 (20.9)	135 (16.1)	247 (13.9)		1029 (21.2)	132 (16.0)	233 (14.7)		1754 (21.1)	267 (16.1)	480 (14.3)	
Current	2583 (74.5)	675 (80.7)	1451 (82.0)		3012 (62.2)	598 (72.6)	1177 (74.2)		5595 (67.3)	1273 (76.6)	2628 (78.3)	
Body-mass Index (kg/m2)	26.9	26.5	25.9	**<0.0001**	26.7	25.5	24.9	**<0.0001**	26.8	26.0	25.4	**<0.0001**
Median (1Q – 3Q)	(24.3 – 29.9)	(24.0 – 29.2)	(23.9 – 28.5)		(23.7 –30.4)	(22.8 – 29.0)	(22.7 –28.0)		(23.9 – 30.2)	(23.4 – 29.1)	(23.3 –28.3)	
Waist circumference (cm)	96.0	94.6	91.6	**<0.0001**	87.3	85.0	82.9	**<0.0001**	91.2	90.0	88.2	**<0.0001**
Median (1Q – 3Q)	(88.5 –104)	(88.0 – 102)	(85.4 – 99.3)		(79.8 –96.5)	(77.5 – 93.7)	(75.9 –91.0)		(82.8 –100.)	(81.5 – 98.5)	(80.2 –96.3)	
Hypertension (%)	1500 (43.3)	333 (39.8)	546 (30.8)	**<0.0001**	1631 (33.6)	239 (28.9)	393 (24.7)	**<0.0001**	3131 (37.7)	572 (34.4)	939 (27.9)	**<0.0001**
Diabetes (%)	744 (21.5)	157 (18.8)	216 (12.2)	**<0.0001**	658 (13.6)	104 (12.6)	158 (9.9)	**0.0008**	1402 (16.9)	261 (15.7)	374 (11.1)	**<0.0001**
Dyslipidemia (%)	1619 (46.7)	393 (47.0)	813 (45.9)	0.8354	2149 (44.3)	388 (47.0)	687 (43.2)	0.2077	3768 (45.3)	781 (47.0)	1500 (44.6)	0.2970
Depression (%)	94 (2.7)	7 (0.8)	30 (1.7)	**<0.0001**	306 (6.3)	26 (3.1)	51 (3.2)	**<0.0001**	400 (4.8)	33 (2.0)	81 (2.4)	**<0.0001**

Values are expressed as n (%) for categorical variables and as median (1^st^–3rd quartiles) for continuous variables.

Comparisons across LTPA categories (inactive, insufficiently active, and active) were performed using the Chi-Square test for categorical variables and the Kruskal-Wallis test for continuous variables. p<0.05 indicates statistically significant differences among LTPA categories.

Baseline characteristics by CPA (Table 2). Participants with higher CPA were more frequently Black or mixed race and tended to have lower education and income (all p < 0.0001). Median age increased slightly across CPA levels (from 50 to 52 years; p < 0.0001). BMI and waist circumference were lower among active commuters. Hypertension prevalence was higher among active women and in the total sample (p < 0.0001). Differences in depression were small but statistically significant (p=0.002–0.041). No consistent differences were observed for diabetes or dyslipidemia.

**Table 2:** Sociodemographic and clinical characteristics by Commuting Physical Activity (CPA) levels in men, women, and overall sample.

	Men (n = 6074)				Women (n = 7266)				Overall (n = 13340)			
	Inactive (n=1518)	Insufficiently active (n=2377)	Active (n=2179)	p	Inactive (n=2032)	Insufficiently active (n=2894)	Active (n=2340)	p	Inactive (n=3550)	Insufficiently active (n=5271)	Active (n=4519)	p
Age (years)	50	51	51	**0.0012**	50	51	52	**<0.0001**	50	51	52	**<0.0001**
Median (1Q – 3Q)	(44.0 – 57.0)	(45.0 – 58.0)	(45.0 – 59.0)		(44.0 –57.0)	(45.0 – 58.0)	(46.0 –59.0)		(44.0 – 57.0)	(45.0 – 58.0)	(46.0 –59.0)	
Race (%)				**<0.0001**				**<0.0001**				**<0.0001**
White	857 (56.5)	1334 (56.1)	1068 (49.0)		1192 (58.7)	1480 (51.1)	1114 (47.6)		2049 (57.7)	2814 (53.4)	2182 (48.3)	
Mixed	424 (27.9)	687 (28.9)	667 (30.6)		513 (25.2)	780 (27.0)	617 (26.4)		937 (26.4)	1467 (27.8)	1284 (28.4)	
Black	168 (11.1)	257 (10.8)	354 (16.2)		237 (11.7)	499 (17.2)	490 (20.9)		405 (11.4)	756 (14.4)	844 (18.7)	
Others	69 (4.5)	99 (4.2)	90 (4.1)		90 (4.4)	135 (4.7)	119 (5.1)		159 (4.5)	234 (4.4)	209 (4.6)	
Education (%)				**<0.0001**				**<0.0001**				**<0.0001**
Less than high school	141 (9.3)	287 (12.1)	337 (15.5)		99 (4.9)	232 (8.0)	198 (8.5)		240 (6.8)	519 (9.9)	535 (11.8)	
High school and some College	421 (27.7)	706 (29.7)	891 (40.9)		572 (28.1)	997 (34.5)	1023 (43.7)		994 (28.0)	1703 (32.3)	1914 (42.4)	
Complete College or more	956 (63.0)	1384 (58.2)	951 (43.6)		1361 (67.0)	1665 (57.5)	1119 (47.8)		2317 (65.2)	3049 (57.8)	2070 (45.8)	
Average family monthly net income (%)				**<0.0001**				**<0.0001**				**<0.0001**
**<US$ 1245**	242 (16.0)	498 (21.0)	713 (32.9)		303 (15.0)	712 (24.7)	756 (32.5)		545 (15.4)	1210 (23.0)	1469 (32.7)	
US$ 1245 – 3320	621 (40.9)	962 (40.5)	945 (43.6)		932 (46.0)	1366 (47.3)	1117 (47.9)		1553 (43.8)	2328 (44.3)	2062 (45.8)	
**>US$ 3320**	654 (43.1)	914 (38.5)	511 (23.5)		790 (39.0)	809 (28.0)	456 (19.6)		1444 (40.8)	1723 (32.7)	967 (21.5)	
Smoking (%)				**<0.0001**				**<0.0001**				**<0.0001**
Never	756 (49.8)	1282 (53.9)	1061 (48.7)		1203 (59.2)	1905 (65.8)	1457 (62.3)		1959 (55.2)	3187 (60.5)	2518 (55.7)	
Past	553 (36.4)	814 (34.3)	757 (34.7)		594 (29.2)	664 (23.0)	586 (25.0)		1147 (32.3)	1478 (28.0)	1343 (29.7)	
Current	209 (13.8)	281 (11.8)	361 (16.6)		235 (11.6)	325 (11.2)	297 (12.7)		444 (12.5)	606 (11.5)	658 (14.6)	
Alcohol intake				**0.0025**				**<0.0001**				**<0.0001**
Never	55 (3.6)	104 (4.4)	98 (4.5)		287 (14.1)	432 (14.9)	354 (15.2)		342 (9.6)	536 (10.2)	452(10.0)	
Past	277 (18.3)	384 (16.1)	446 (20.5)		329 (16.2)	554 (19.2)	511 (21.9)		606 (17.1)	938 (17.8)	957 (21.2)	
Current	1186 (78.1)	1889 (79.5)	1634 (75.0)		1414 (69.7)	1906 (65.9)	1467 (62.9)		2600 (73.3)	3795 (72.0)	3101 (68.8)	
Body-mass Index (kg/m^2^)	27.0	26.7	26.1	**<0.0001**	26.1	25.9	26.3	**0.0123**	26.5	26.3	26.2	**0.0014**
Median (1Q – 3Q)	(24.3 – 29.9)	(24.2 – 29.3)	(23.8 – 29.0)		(23.4 –30.1)	(23.2 – 29.5)	(23.5 –29.9)		(23.8 – 30.0)	(23.6 – 29.4)	(23.6 –29.4)	
Waist circumference (cm)	96.2	94.8	93.4	**<0.0001**	86.3	85.6	86.2	**0.0014**	91.0	89.8	90.2	**0.0088**
Median (1Q – 3Q)	(88.6 – 104)	(87.5 – 102)	(86.5 – 100)		(78.7 –95.2)	(77.8 – 94.4)	(79.1 –95.2)		(82.2 – 100)	(81.5 – 99.2)	(82.0 –98.5)	
Hypertension (%)	571 (37.6)	952 (40.1)	856 (39.3)	0.3127	548 (27.0)	899 (31.1)	816 (34.9)	**<0.0001**	1119 (31.5)	1851 (35.1)	1672 (37.0)	**<0.0001**
Diabetes (%)	280 (18.5)	432 (18.2)	405 (18.6)	0.9342	238 (11.7)	357 (12.3)	325 (13.9)	0.0779	518 (14.6)	789 (15.0)	730 (16.2)	0.1156
Dyslipidemia (%)	729 (48.1)	1122 (47.2)	974 (44.7)	0.0914	895 (44.0)	1254 (43.3)	1075 (45.9)	0.1610	1624 (45.8)	2376 (45.1)	2049 (45.3)	0.8233
Depression (%)	45 (3.0)	43 (1.8)	43 (2.0)	0.0407	123 (6.1)	130 (4.5)	130 (5.6)	0.0411	168 (4.7)	173 (3.3)	173 (3.8)	0.0024

Values are expressed as n (%) for categorical variables and as median (1^st^–3^rd^ quartiles) for continuous variables.

Comparisons across CPA categories (inactive, insufficiently active, and active) were performed using the Chi-Square test for categorical variables and the Kruskal-Wallis test for continuous variables. p<0.05 indicates statistically significant differences among CPA categories.

At Table 3, in the fully adjusted model (Model 2), LTPA inactivity was associated with poorer performance in verbal fluency (β = -0.07; 95% CI -0.10 to -0.04), executive function measured by the TMT-B (β = -0.10; 95% CI -0.13 to -0.06), and global cognition (β = -0.06; 95% CI -0.08 to -0.03) in both sexes. The association with memory was modest and borderline (β = -0.03; 95% CI -0.06 to -0.0003). After additional adjustment for occupational status (Model 3), estimates attenuated slightly but remained significant for verbal fluency, TMT-B, and global cognition, while the borderline association for memory persisted.

**Table 3:** Linear Regression (Beta [β] and 95% Confidence Intervals [95% CI]) for the association of crude and adjusted models between LTPA and cognitive test performance in men, women, and overall sample, using the active group as reference.

LTPA		Crude	Model 1	Model 2	Model 3 (occupation)
Men		β [95%CI]	β [95%CI]	β [95%CI]	β [95%CI]
Memory score	Inactive	**-0.17 [-0.22; -0.12]**	-0.04 [-0.09; 0.003]	-0.03 [-0.07; 0.02]	-0.02 [-0.07; 0.02]
	Insufficiently active	-0.06 [-0.12; 0.01]	0.03 [-0.04; 0.09]	0.03 [-0.03; 0.09]	0.04 [-0.03; 0.10]
Verbal Fluency score	Inactive	**-0.21 [-0.25; -0.16]**	**-0.05 [-0.09; -0.01]**	**-0.05 [-0.09; -0.002]**	-0.04 [-0.09; 0.002]
	Insufficiently active	**-0.08 [-0.15; -0.01]**	0.002 [-0.06; 0.06]	0.003 [-0.06; 0.06]	0.003 [-0.06; 0.06]
Trail score	Inactive	**-0.34 [-0.40; -0.28]**	**-0.10 [-0.15; -0.05]**	**-0.10 [-0.15; -0.05]**	**-0.09 [-0.13; -0.04]**
	Insufficiently active	**-0.15 [-0.24; -0.07]**	-0.02 [-0.09; 0.05]	-0.02 [-0.09; 0.05]	-0.02 [-0.09; 0.05]
Global score	Inactive	**-0.21 [-0.25; -0.17]**	**-0.05 [-0.09; -0.02]**	**-0.05 [-0.08; -0.01]**	**-0.04 [-0.07; -0.01]**
	Insufficiently active	**-0.08 [-0.13; -0.02]**	0.01 [-0.03; 0.06]	0.01 [-0.03; 0.06]	0.02 [-0.03; 0.06]
**Women**					
Memory score	Inactive	**-0.15 [-0.19; -0.10]**	**-0.05 [-0.08; -0.01]**	-0.03 [-0.07; 0.01]	-0.03 [-0.07; 0.01]
	Insufficiently active	0.001 [-0.06; 0.06]	0.01 [-0.04; 0.07]	0.02 [-0.04; 0.07]	0.02 [-0.04; 0.08]
Verbal Fluency score	Inactive	**-0.26 [-0.31; -0.21]**	**-0.09 [-0.13; -0.05]**	**-0.09 [-0.13; -0.05]**	**-0.08 [-0.12; -0.04]**
	Insufficiently active	-0.01 [-0.08; 0.05]	-0.01 [-0.07; 0.06]	-0.004 [-0.06; 0.06]	-0.01 [-0.07; 0.05]
Trail score	Inactive	**-0.31 [-0.36; -0.26]**	**-0.10 [-0.15; -0.05]**	**-0.10 [-0.15; -0.05]**	**-0.08 [-0.13; -0.04]**
	Insufficiently active	-0.06 [-0.14; 0.02]	-0.03 [-0.10; 0.04]	-0.03 [-0.10; 0.04]	-0.03 [-0.09; 0.04]
Global score	Inactive	**-0.21 [-0.24; -0.18]**	**-0.07 [-0.10; -0.04]**	**-0.06 [-0.09; -0.03]**	**-0.06 [-0.09; -0.03]**
	Insufficiently active	-0.01 [-0.07; 0.04]	0.0001 [-0.04; 0.04]	0.003 [-0.04; 0.05]	0.002 [-0.04; 0.05]
**Overall**					
Memory score	Inactive	**-0.12 [-0.16; -0.09]**	**-0.05 [-0.08; -0.02]**	**-0.03 [-0.06; -0.004]**	**-0.03 [-0.06; -0.0003]**
	Insufficiently active	-0.02 [-0.07; 0.03]	0.02 [-0.03; 0.06]	0.02 [-0.02; 0.06]	0.03 [-0.02; 0.07]
Verbal Fluency score	Inactive	**-0.22 [-0.26; -0.19]**	**-0.07 [-0.10; -0.04]**	**-0.07 [-0.10; -0.04]**	**-0.06 [-0.10; -0.03]**
	Insufficiently active	-0.05 [-0.09; 0.003]	-0.002 [-0.04; 0.04]	-0.001 [-0.04; 0.04]	-0.003 [-0.05; 0.04]
Trail score	Inactive	**-0.32 [-0.36; -0.28]**	**-0.10 [-0.13; -0.07]**	**-0.10 [-0.13; -0.06]**	**-0.08 [-0.12; -0.05]**
	Insufficiently active	**-0.11 [-0.17; -0.05]**	-0.03 [-0.08; 0.02]	-0.03 [-0.08; 0.02]	-0.03 [-0.07; 0.02]
Global score	Inactive	**-0.19 [-0.22; -0.16]**	**-0.06 [-0.09; -0.04]**	**-0.06 [-0.08; -0.03]**	**-0.05 [-0.07; -0.03]**
	Insufficiently active	**-0.04 [-0.08; -0.01]**	0.003 [-0.03; 0.03]	0.01 [-0.03; 0.04]	0.01 [-0.02; 0.04]

Crude: No adjustment; Model 1: Adjusted by age, sex, self-reported race and education attainment; Model 2: Model 1 + BMI, smoking, hypertension, diabetes, depression; Model 3: Model 2 more and occupational status.

At Table 4, before adjustment for occupational status (Model 2), CPA showed associations in the opposite direction. Among men, inactivity was associated with better memory (β = 0.05; 95% CI: 0.001 to 0.10; borderline) and better executive function on the TMT-B (β = 0.06; 95% CI: 0.01 to 0.12). Men classified as insufficiently active also showed a borderline association with TMT-B (β = 0.05; 95% CI: 0.003 to 0.10). Among women, inactivity was associated with better TMT-B performance (β = 0.10; 95% CI: 0.05 to 0.15). In the overall sample, CPA inactivity was positively associated with executive function (β = 0.08; 95% CI: 0.05 to 0.12).

**Table 4:** Linear Regression (Beta [β] and 95% Confidence Intervals [95% CI]) for the association of crude and adjusted models between Commuting Physical Activity (CPA) and cognitive test performance in men, women, and overall sample, using the active group as reference.

Commuting Physical Activity		Crude	Model 1	Model 2	Model 3 (occupation)
Men		β [95%CI]	β [95%CI]	β [95%CI]	β [95%CI]
Memory score	Inactive	**0.17 [0.11; 0.22]**	0.05 [-0.01; 0.10]	**0.05 [0.001; 0.10]**	0.04 [-0.01; 0.10]
	Insufficiently active	**0.11 [0.06; 0.16]**	0.03 [-0.02; 0.07]	0.02 [-0.02; 0.07]	0.01 [-0.03; 0.06]
Verbal Fluency score	Inactive	**0.13 [0.08; 0.19]**	-0.03 [-0.08; 0.02]	-0.03 [-0.08; 0.02]	-0.03 [-0.08; 0.02]
	Insufficiently active	**0.10 [0.05; 0.15]**	-0.01 [-0.06; 0.03]	-0.01 [-0.05; 0.03]	-0.01 [-0.05; 0.03]
Trail score	Inactive	**0.30 [0.24; 0.37]**	**0.06 [0.004; 0.12]**	**0.06 [0.01; 0.12]**	0.05 [-0.01; 0.10]
	Insufficiently active	**0.22 [0.16; 0.28]**	**0.05 [0.001; 0.10]**	**0.05 [0.003; 0.10]**	0.04 [-0.01; 0.09]
Global score	Inactive	**0.18 [0.14; 0.22]**	0.02 [-0.01; 0.06]	0.03 [-0.01; 0.06]	0.02 [-0.02; 0.05]
	Insufficiently active	**0.12 [0.08; 0.16]**	0.02 [-0.02; 0.05]	0.02 [-0.02; 0.05]	0.01 [-0.02; 0.04]
**Women**					
Memory score	Inactive	**0.08 [0.04; 0.13]**	-0.02 [-0.06; 0.02]	-0.02 [-0.06; 0.02]	-0.02 [-0.06; 0.02]
	Insufficiently active	**0.05 [0.01; 0.09]**	-0.01 [-0.04; 0.03]	-0.01 [-0.05; 0.03]	-0.01 [-0.05; 0.02]
Verbal Fluency score	Inactive	**0.19 [0.15; 0.24]**	0.04 [-0.002; 0.09]	0.04 [-0.01; 0.08]	0.03 [-0.02; 0.07]
	Insufficiently active	**0.06 [0.02; 0.11]**	-0.01 [-0.05; 0.03]	-0.01 [-0.05; 0.03]	-0.02 [-0.06; 0.02]
Trail score	Inactive	**0.33 [0.27; 0.39]**	**0.11 [0.06; 0.15]**	**0.10 [0.05; 0.15]**	**0.08 [0.04; 0.13]**
	Insufficiently active	**0.12 [0.07; 0.17]**	0.01 [-0.03; 0.06]	0.01 [-0.03; 0.06]	-0.002 [-0.05; 0.04]
Global score	Inactive	**0.16 [0.13; 0.20]**	0.02 [-0.01; 0.05]	0.02 [-0.01; 0.05]	0.01 [-0.02; 0.04]
	Insufficiently active	**0.06 [0.03; 0.10]**	-0.004 [-0.03; 0.02]	-0.01 [-0.03; 0.02]	-0.01 [-0.04; 0.02]
**Overall**					
Memory score	Inactive	**0.14 [0.10; 0.17]**	0.01 [-0.02; 0.04]	0.01 [-0.02; 0.05]	0.01 [-0.02; 0.04]
	Insufficiently active	**0.08 [0.05; 0.12]**	0.01 [-0.02; 0.04]	0.01 [-0.02; 0.04]	0.004 [-0.03; 0.03]
Verbal Fluency score	Inactive	**0.17 [0.14; 0.21]**	0.01 [-0.02; 0.04]	0.01 [-0.02; 0.04]	0.0002 [-0.03; 0.03]
	Insufficiently active	**0.08 [0.05; 0.11]**	-0.01 [-0.04; 0.02]	-0.01 [-0.04; 0.02]	-0.01 [-0.04; 0.02]
Trail score	Inactive	**0.32 [0.28; 0.36]**	**0.09 [0.05; 0.12]**	**0.08 [0.05; 0.12]**	**0.07 [0.03; 0.10]**
	Insufficiently active	**0.16 [0.12; 0.20]**	0.03 [-0.001; 0.06]	**0.03 [0.001; 0.07]**	0.02 [-0.02; 0.05]
Global score	Inactive	**0.18 [0.15; 0.21]**	0.02 [-0.002; 0.05]	**0.02 [0.001; 0.05]**	0.02 [-0.01; 0.04]
	Insufficiently active	**0.10 [0.07; 0.12]**	0.01 [-0.02; 0.03]	0.01 [-0.015; 0.03]	-0.002 [-0.02; 0.02]

Crude: No adjustment; Model 1: Adjusted by age, sex, self-reported race and education attainment; Model 2: Model 1 + BMI, smoking, hypertension, diabetes, depression; Model 3: Model 2 more and occupational status.

After additional adjustment for occupational status (Model 3), CPA associations attenuated. In men, previously observed associations with memory and TMT-B lost statistical significance. In women, the association with TMT-B remained significant (β = 0.08; 95% CI: 0.04 to 0.13). In the overall sample, the positive association between CPA inactivity and TMT-B also remained significant, although with reduced magnitude (β = 0.07; 95% CI: 0.03 to 0.10).

## Discussion

The findings of this study indicate that the association between PA and cognitive performance varies according to the domain in which the activity is performed. LTPA showed the most consistent relationship with cognitive outcomes, with physical inactivity being associated with lower performance in multiple cognitive domains, even after adjusting for relevant covariates. In comparison, CPA showed associations with executive function, particularly in analyses where inactive individuals performed better than their active counterparts. When total PA was examined, the associations with cognition were weaker and more limited, suggesting that the type and context of PA may play a more relevant role than the overall volume. These differences reinforce the importance of evaluating PA patterns in a disaggregated manner when examining cognitive outcomes. It is important to note that being a cross-sectional study, it is possible to conclude about associations, but not causality.

The present study analyzed the associations between PA in different domains and cognitive performance, with attention to sex-specific differences. LTPA showed consistent associations with cognitive outcomes. Individuals classified as inactive in LTPA demonstrated lower performance in memory, verbal fluency, executive function, and global cognitive score, both in sex-stratified analyses and in the total sample. These results are in line with findings from prospective studies indicating that regular LTPA during midlife is associated with a reduced risk of dementia and cognitive decline. Previous studies have confirmed these associations using various cognitive outcomes and follow-up periods.^25^^,^^42^, ^43^, ^44^, ^45^, ^46^ Although most of these studies used longitudinal designs, the consistency of the evidence supports the role of LTPA in cognitive health.

In contrast, CPA showed a more specific pattern of association. In this study, CPA was inversely associated with performance in the TMT-B, which evaluates executive function. This association was observed among men and in the total sample. No significant associations were found between commuting activity and memory, verbal fluency, or global cognitive performance. These results suggest that PA during commuting may relate differently to specific cognitive domains, but the direction of the association was not favorable. Similar suppressor effects on executive function measured via the TMT-B have been observed in previous research, where the direct association between PA and executive function was also negative after controlling for processing speed.^47^ Moreover, other studies have found no significant associations between active commuting and perceived memory, concentration, or cognitive performance,^48^ reinforcing the domain-specific and non-favorable nature of these effects.

The finding related to executive function is consistent with studies indicating that moderate-intensity PA can improve or maintain functions such as attention, flexibility, and processing speed. However, the context in which CPA occurs should also be considered. Urban environments are often characterized by exposure to traffic, noise, and air pollution, which may attenuate or modify the effects of PA on brain health. Although not directly assessed in this study, previous analyses from the ELSA‒Brazil cohort and other studies have linked commuting in polluted areas with adverse cardiovascular and metabolic outcomes.^7^^,^^49^^,^^50^ These environmental factors have been proposed as relevant variables in the interaction between PA and health outcomes.^51^, ^52^, ^53^, ^54^

The authors hypothesize that the unexpected association of CPA and worse cognitive performance is explained by the fact that urban environments are often characterized by exposure to traffic, noise, and air pollution, which may attenuate or antagonize the effects of physical activity on health. The practice of physical activity is always important, as sedentary behavior is a known risk factor for cardiovascular and other chronic diseases. However, the present findings for the association between TMT-B and CPA lost their significance after adjustment for occupational status. If any association between TMT-B and CPA really exists, it would likely be explained by environmental conditions rather than by the practice of physical activity per se. The loss of significance after further adjustment for occupational status may indicate the influence of reverse causation.

Previous evidence has shown that LTPA involves voluntary engagement, use of large muscle groups, and opportunities to rest, which may contribute to more favorable effects on systemic and brain function.^55^ In contrast, CPA is often performed under time constraints, in less favorable settings, and may not offer the same physiological and psychological benefits. This distinction has been highlighted in studies differentiating the effects of PA according to its context.^56^ Although that study focused on sickness absence rather than cognition, it contributed to the understanding that the domain of PA matters in determining health outcomes.

To date, there is a lack of studies that examine CPA as an independent exposure for cognitive performance. One study using data from the CAIDE cohort analyzed both commuting and LTPA but found no significant association between commuting and dementia risk after multivariable adjustment.^57^ In that study, only LTPA showed beneficial effects. Another study by^23^ found that only LTPA mediated the association between education and cognitive function, while CPA showed no mediation effect. These findings suggest that LTPA may have a more robust relationship with cognition compared to activity performed during transportation.

In the present analysis, no interaction by sex was observed in the associations between LTPA and cognitive outcomes. Both men and women who were inactive in LTPA presented lower cognitive performance. For CPA, although a positive association with executive function was observed, especially among men, the differences by sex were not statistically robust. Few previous studies have analyzed PA and cognition stratified by sex, which limits comparisons.^58^ Nonetheless, the current findings reinforce that LTPA may offer cognitive benefits across demographic groups.

This study has several limitations that should be acknowledged. Its cross-sectional design precludes any inference of causality between PA and cognitive performance. Although the authors showed some associations, it is impossible to show that the pattern of practice of physical activity may influence cognitive performance, but they cannot exclude that the worse or better cognitive performance may also influence the pattern of practice of physical activity. Therefore, the study shows association but not causality. Even after so many adjustments, it is important to note that some kind of residual confounder is still possible. The study has no information about environmental risk factors such as air pollution. Therefore, it was not possible to include these variables in the multivariable models, which may limit the interpretation of associations related to CPA.

The measurement of PA is a limitation of this study. Self-reported information obtained from the IPAQ is subject to recall and desirability bias. The long version of the instrument, restricted to the leisure-time and commuting modules, was adopted to ensure comparability with studies conducted in Brazil and other Latin American countries. This approach is supported by the review by Hallal et al. (2010), which concluded that these two modules of the long IPAQ are the most appropriate for quantifying physical activity in epidemiological research in the region. The categorization into active, insufficiently active, and inactive groups followed WHO recommendations.^59^

Nonetheless, this study has notable strengths. It draws on data from ELSA‒Brazil, a large, multiethnic cohort based in a middle-income country, providing a relevant contrast to prior studies conducted predominantly in high-income contexts. The sample was rigorously recruited, and data were collected under strict quality control. Furthermore, standardized cognitive assessments were used, and the study examined LTPA and CPA separately, stratified by sex, allowing for a more nuanced analysis of the associations with cognitive function.

## Conclusion

In conclusion, LTPA inactivity was consistently associated with poorer performance in verbal fluency, executive function, and global cognition in both men and women. The positive association between CPA and TMT-B observed among men lost significance after adjustment for occupational status. These cross-sectional findings indicate that the relationship between physical activity and cognition differs by domain and should be investigated in longitudinal analyses.

## Data availability statement

The data that support the findings of this study are derived from the Brazilian Longitudinal Study of Adult Health (ELSA‒Brazil). Due to privacy, ethical, and institutional restrictions, these data are not publicly available. Data access requests can be made to the corresponding author upon reasonable request.

## Note on reference count

The manuscript includes 59 references due to its multidisciplinary nature, combining two physical activity domains, multiple cognitive outcomes, and methodological sources from the ELSA‒Brazil cohort. All citations are directly relevant to the study’s design and interpretation and are essential for maintaining scientific rigor and contextual accuracy.

## Authors’ contributions

Amanda M. Pádua: Conceptualization (Equal); Data curation (Equal); Formal analysis (Equal); Investigation (Lead); Methodology (Equal); Software (Equal); Validation (Equal); Visualization (Lead); Writing-original draft (Lead); Writing-review & editing (Equal). Claudia K. Suemoto: Software (Equal); Validation (Equal); Visualization (Equal); Writing-review & editing (Equal). Patrícia Fonseca Estrada (Corresponding Author): Conceptualization (Equal); Data curation (Equal); Methodology (Equal); Software (Equal); Validation (Equal); Visualization (Equal); Writing-review & editing (Equal). Marcos R. N. Cavalcante: Data curation (Equal); Investigation (Equal); Software (Equal); Writing-review & editing (Equal). Alessandra C. Goulart: Data curation (Equal); Software (Equal); Validation (Equal); Writing-review & editing (Equal). Paulo Andrade Lotufo: Funding acquisition (Lead); Investigation (Equal); Project administration (Lead); Validation (Equal); Visualization (Equal); Writing-review & editing (Equal). Itamar de Souza Santos: Software (Equal); Validation (Equal); Visualization (Equal); Writing-review & editing (Equal). Isabela M. Bensenor: Conceptualization (Lead); Data curation (Lead); Funding acquisition (Lead); Methodology (Equal); Project administration (Lead); Resources (Equal); Supervision (Lead); Validation (Equal); Visualization (Equal); Writing-review & editing (Lead).

## Funding

The ELSA‒Brazil baseline study and the 8-year follow-up assessment were supported by the Brazilian Ministry of Health (Science and Technology Department), the Brazilian Ministry of Science and Technology (*Financiadora de Estudos e Projetos*), and the National Research Council (CNPq). Grants related to the baseline assessment: 01 06 0010.00 RS, 01 06 0212.00 BA, 01 06 0300.00 ES, 01 06 0278.00 MG, 01 06 0115.00 SP, and 01 06 0071.00 RJ. Grants related to the follow-up assessment: 01 06 0212-00 BA, 01 06 0300-00 ES, 01 06 0278-00 MG, 01 06 0010-00 RS, 01 06 0071-00 RJ, and 01 06 0115-00 SP. This study was also supported by FAPESP – Fundação de Amparo à Pesquisa do Estado de São Paulo (doctoral scholarship of P.F.E., process 2024/04191-0). A.M.P. and I.M.B. are recipients of scholarships from the National Research Council (CNPq).

## Declaration of competing interest

The authors declare no conflicts of interest.
